# The Probiotic *Bifidobacterium breve* B632 Inhibited the Growth of *Enterobacteriaceae* within Colicky Infant Microbiota Cultures

**DOI:** 10.1155/2014/301053

**Published:** 2014-08-28

**Authors:** Marta Simone, Caterina Gozzoli, Andrea Quartieri, Giuseppe Mazzola, Diana Di Gioia, Alberto Amaretti, Stefano Raimondi, Maddalena Rossi

**Affiliations:** ^1^Dipartimento di Scienze della Vita, Università di Modena e Reggio Emilia, Viale G. Campi 183, 41125 Modena, Italy; ^2^Dipartimento di Scienze Agrarie, Università di Bologna, Viale Fanin 44, 40127 Bologna, Italy

## Abstract

Infant colic is a common gastrointestinal disorder of newborns, mostly related to imbalances in the composition of gut microbiota and particularly to the presence of gas-producing coliforms and to lower levels of Bifidobacteria and Lactobacilli. Probiotics could help to contain this disturbance, with formulations consisting of *Lactobacillus* strains being the most utilized. In this work, the probiotic strain *Bifidobacterium breve* B632 that was specifically selected for its ability to inhibit gas-producing coliforms, was challenged against the *Enterobacteriaceae* within continuous cultures of microbiota from a 2-month-old colicky infant. As confirmed by RAPD-PCR fingerprinting, *B. breve* B632 persisted in probiotic-supplemented microbiota cultures, accounting for the 64% of Bifidobacteria at the steady state. The probiotic succeeded in inhibiting coliforms, since FISH and qPCR revealed that the amount of *Enterobacteriaceae* after 18 h of cultivation was 0.42 and 0.44 magnitude orders lower (*P* < 0.05) in probiotic-supplemented microbiota cultures than in the control ones. These results support the possibility to move to another level of study, that is, the administration of *B. breve* B632 to a cohort of colicky newborns, in order to observe the behavior of this strain *in vivo* and to validate its effect in colic treatment.

## 1. Introduction

In the first hours of life, the germ-free gastrointestinal tract of newborns is colonized by microorganisms deriving from the mother and from the environment, with the establishment of a microbial community that will evolve into one of the most complex microbial ecosystems [1]. The maintenance of a correct balance of gut bacterial population is extremely important since microbiota performs a variety of activities and functions that deeply influence the health status of the host, such as the metabolism of nondigestible compounds with supply of short chain fatty acids, vitamin biosynthesis, the regulation of immune system, and the prevention of pathogen colonization [2, 3].

Despite the fact that increasing information about microbiota composition in adults is arising from metagenomics and other culture-independent approaches, the dynamics of initial colonization and evolution of the bacterial community during the first days of life are poorly understood so far [4]. In newborns, microbiota composition is variable and unstable, and the establishment of the intestinal microbiota is highly dependent on many factors, such as the mode of birth, breast or formula feeding, and antibiotic intake [5–7]. Furthermore, factors affecting the tropism and host-microbe interactions, such as intestinal pH, body temperature, bile acids, peristalsis, mucosal immune response receptors, and internal synergy, exert a pivoting role in shaping the composition of bacterial population [8, 9]. Initially, culturing studies indicated that the pioneer bacteria colonizing the digestive tract of newborns are Enterobacteriaceae and Gram-positive cocci (e.g.,* Streptococcus*,* Staphylococcus*), which lower the redox potential and generate an anoxic environment, favorable for the establishment of strictly anaerobic bacteria, such as Bacteroidetes,* Bifidobacterium*, and Clostridiales [8, 10].* Bifidobacteria* are generally reported to prevail in the gut microbiota of naturally delivered breast-fed infants after a few days, at the expenses of Enterobacteriaceae and facultative aerobes [11]. However, culture independent investigations have provided evidence that infant colonization may be much more complex, since it may be primed by anaerobes as well (e.g. Clostridiales) and* Bifidobacteria* may not be among the first colonizers or may remain a numerical minority [12].

Infant colic is a common functional gastrointestinal disorder of newborns, characterized by long bouts of crying and hard-to-relieve behavior [13]. Crying peaks range between 6 and 12 weeks of age and cause considerable concern and distress to parents. The pathogenesis of infant colic is not well understood, and several underlying causes have been suggested [13]. Among them, the relationship between colonic microbiota and this disorder is emerging as a major determinant. Culturing studies revealed higher counts of Gram-negative bacteria and a less numerous population of* Lactobacilli* and* Bifidobacteria* in the feces of colicky infants compared with healthy infants [14]. Molecular global investigation of the microbiota composition through phylogenetic microarray analysis demonstrated that gut microbiota differentiate much more slowly in colicky infants than in healthy ones and that colic correlated positively with the presence of specific genera of Gammaproteobacteria (such as* Escherichia*,* Klebsiella*,* Serratia*,* Vibrio*,* Yersinia*, and* Pseudomonas*) and negatively with bacteria belonging to the Bacteroidetes and Firmicutes [15, 16]. Consistently, it is known that Enterobacteriaceae, such as bacteria belonging to* Escherichia* and* Klebsiella*, produce gas from mixed acid fermentation and proinflammatory lipopolysaccharides, both these mechanisms being proposed to favor colic [17, 18].

The microbiota of colicky infants also presents lower amounts of* Bifidobacteria* and* Lactobacilli*, which are known to be anti-inflammatory and to exert various healthy properties [19–21]. The intake of probiotic* Lactobacilli* during the first months of life can contribute to containing colic [22, 23]. On the contrary,* in vivo* studies utilizing probiotic* Bifidobacteria* for the treatment of colic are lacking. The strain* Bifidobacterium breve* B632 possesses antimicrobial activity against gas-producing coliforms isolated from the stools of infants suffering from colic [24].

In order to obtain preliminary results that could support an* in vivo* trial, the present study challenged* B. breve* B632 against the Enterobacteriaceae within cultures of microbiota from a 2-month-old colicky infant. A continuous culture fermentation simulating the gut microbiota of a colicky infant was performed to examine the time-course of* E. coli* and Enterobacteriaceae populations.

## 2. Methods

### 2.1. Chemicals and Bacterial Strain

All the chemicals were supplied by Sigma (Stenheim, Germany), unless otherwise stated.* Bifidobacterium breve* B632 was obtained from BUSCoB strain collection (Scardovi Collection of* Bifidobacteria*, Dept. of Agro-Environmental Science and Technology, University of Bologna, Italy). The strain was accepted for deposit by DSMZ for patent purposes and named* B. breve* DSMZ 24706. It was cultured anaerobically at 37°C in Lactobacilli MRS broth (BD Difco, Sparks, USA) containing 0.5 g/L L-cysteine hydrochloride (hereinafter called MRS).

### 2.2. Cultures of Gut Microbiota

The cultures of gut microbiota were performed in a microbiota medium MM [25], where the carbon source was substituted with 6.0 g/L of a mixture of galactooligosaccharides (GOS, Domo Vivinal, Needseweg, The Netherlands) and fructooligosaccharides (FOS, Beneo-Orafti P95, Oreye, Belgium). The mixture was composed of 90% GOS and 10% FOS (w/w), in agreement with the composition of prebiotic infant formula [26]. Oligosaccharides were filter-sterilized (0.22 *μ*m) and added to the medium after autoclaving.

Fresh feces from a breast-fed colicky infant, born by natural delivery and not treated with antibiotics or probiotics, were utilized to prepare the inoculum for single-stage continuous cultures. Inoculum preparation was performed in anaerobic cabinet under an 85% N_2_, 10% CO_2_, and 5% H_2_ atmosphere. Feces were diluted to the ratio of 1 : 10 (w/v) in MM, supplemented with 10% glycerol (v/v), and stored at −80°C until use.

In control microbiota cultures (MC), 5 mL of fecal suspension was thawed at 37°C and utilized to inoculate bench-top bioreactors (Sixfors V3.01, Infors, Bottmingen, Swiss) containing 250 mL of MM. Fresh MM was fed at the dilution rate of 0.042 h^−1^, corresponding to one turnover per day. The medium was flushed with CO_2_ to maintain anaerobiosis. The culture was kept in anaerobiosis at 37°C, under gentle agitation. Automatic titration with 4 M NaOH maintained pH at 6.5.

In probiotic-supplemented microbiota cultures (PMC), fecal cultures were supplemented with 5.0 E + 7 cfu/mL of* B. breve* B632. Concentrated stock cultures of* B. breve* B632 were supplemented with glycerol (10%, v/v), enumerated onto MRS-agar plates, and stored at −80°C until an appropriate volume was thawed and used for bioreactor inoculation.

Samples from MC and PMC were periodically collected to analyze fermentation products, to examine the microbiota composition, and to enumerate and isolate* bifidobacteria*.

### 2.3. Fluorescent In Situ Hybridization (FISH)

FISH enumeration of total bacteria,* bifidobacteria*, and Enterobacteriaceae was based on the procedure of Harmsen et al. [27], with slight modifications. Culture samples were diluted to the ratio of 1 : 4 with 40 g/L paraformaldehyde and incubated overnight at 4°C. Fixed cells were washed with PBS at pH 7.4 and then dehydrated with PBS-ethanol 1 : 1 solution for 1 h at 4°C. The probes Eub 338, Bif 164, and Enterobact D, were used for total bacteria,* bifidobacteria*, and Enterobacteriaceae, respectively [28]. To perform hybridization, 10 *μ*L of cell suspension, 1 *μ*L of the specific FITC-labeled probe, and 100 *μ*L of hybridization buffer (20 mM TRIS-HCl, 0.9 M NaCl, and 0.1% SDS) were mixed and incubated for 16 h at the temperature specific for each probe [28].

A proper amount of the cell suspension was diluted in 4 mL of washing buffer (20 mM TRIS-HCl, 0.9 M NaCl) and maintained at hybridization temperature for 10 min before being filtered onto 0.2 *μ*m polycarbonate filters (Millipore, Ettenleur, The Netherlands). Filters were mounted on microscope slides with Vectashield (Vector Labs, Burlingame, California). The slides were evaluated with a fluorescence microscope (Eclipse 80i, Nikon Instruments) equipped with mercury arc lamp, FITC specific filter, and digital camera. Depending on the number of fluorescent cells, 30 to 100 microscopic fields were counted and averaged in each slide. Each sample was enumerated in triplicate.

### 2.4. qPCR

Biomass samples from MC and PMC cultures were collected by centrifugation, suspended in PBS (pH 7.8), and extracted with QIAmp DNA Stool Mini Kit (Qiagen, Hilden, Germany) to obtain bacterial gDNA. gDNA was quantified with NanoPhotometer P-Class (Implen GmbH, Munchen, Germany), diluted to 2.5 ng/*μ*L in TE buffer pH 8, and subjected to qPCR analysis with primers targeting Enterobacteriaceae and* Escherichia coli* [29–31]. The set of primers Eco-F (GTTAATACCTTTGCTCATTGA)/Eco-R (ACCAGGGTATCAATCCTGTT) and Ent-F (ATGGCTGTCGTCAGCTCGT)/Ent-R (CCTACTTCTTTTGCAACCCACTC) were used for Enterobacteriaceae and* Escherichia coli*, respectively. The mixture contained 10 *μ*L of SsoFast EvaGreen Supermix, 4 *μ*L of each 2 *μ*M primer, and 2 *μ*L of template. qPCR reaction was carried out with the CFX96 Real-Time System (Bio-Rad Laboratories, Redmond, WA, USA), according to the following protocol: 98°C for 2 min; 45 cycles at 98°C for 0.05 min, 60°C for 0.05 min, and 95°C for 1 min; 65°C for 1 min.

### 2.5. RAPD-PCR Tracing of* Bifidobacterium breve* B632

Fresh culture samples were serially diluted in Wilkins-Chalgren anaerobe broth (Oxoid) in the anaerobic cabinet and plated on RB selective medium, in order to count and isolate* Bifidobacteria* [32]. Genomic DNA was extracted from 200 colonies isolated from the PMC processes, using Instagene matrix (Bio-Rad). RAPD-PCR was carried out in a 15 *μ*L reaction mixture: 10X Dream Taq Buffer (including MgCl_2_ 2 mM), 1.5 *μ*L; dNTPs mixture 0.10 mM, 0,15 *μ*L; 2 *μ*M M13 primer (GAGGGTGGCGGTTCT), 3.75 *μ*L; genomic DNA, 3 *μ*L; and PCR water 5.25 *μ*L. DNA amplification was performed with the following protocol: 94°C for 4 min (1 cycle), 94°C for 1 min, 34°C for 1 min, 72°C for 2 min (45 cycles); 72°C for 7 min (1 cycle). The PCR products were electrophoresed in a 2% agarose gel (25 × 25 cm) for 4 h at a constant voltage (160 V) in TAE buffer (40 mM Tris-acetate, 1 mM EDTA, and pH 8.0). RAPD-PCR profiles were visualized under ultraviolet light after staining with ethidium bromide, followed by digital image capturing. The resulting fingerprints were analyzed by the Gene Directory 2.0 (Syngene, UK) software package. The similarity among digitalized profiles was calculated and a dendrogram was derived with an unweighted pair-group method using arithmetic means (UPGMA).

### 2.6. Analysis of Fermentation Products

The samples were clarified through centrifugation (13,000 ×g, 5 min, 4°C) and filtration (0.22 *μ*m cellulose acetate filter) and stored at −20°C until analyzed. Fermentation products (formic, acetic, lactic, propionic, butyric, and succinic acids and ethanol) were analyzed using a HPLC device (Agilent technologies, Waldbronn, Germany) equipped with refractive index detector and Aminex HPX-87 H ion exclusion column. Isocratic elution was carried out with 0.005 M H_2_SO_4_ at 0.6 mL/min [33].

### 2.7. Statistical Analysis

All values are means of four separate experiments. Comparisons were carried out according to Student's *t*-test. Differences were considered statistically significant for *P* < 0.05.

## 3. Results

### 3.1. Evolution of Fecal Microbial Groups and Fermentation Products

**Figure 1:**
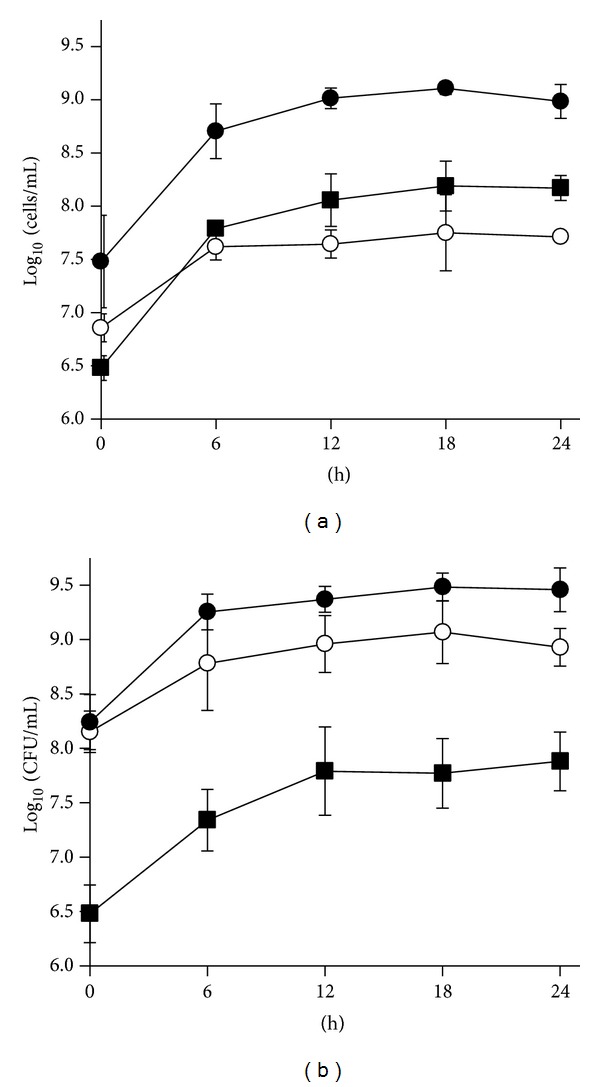
Time-course of total bacteria,* bifidobacteria*, and Enterobacteriaceae in cultures of infant gut microbiota. Eubacteria (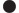),* Bifidobacterium* (⚪), and Enterobacteriaceae (■) were quantified by FISH in control cultures (MC, (a)) and in cultures supplemented with* B*.* breve* B632 (PMC, (b)). Data are means ± SD, *n* = 4.

Single-stage continuous fermentation of the colonic microbiota from a colicky newborn was carried out for 24 h to study whether the addition of* B. breve* B632 could affect the growth of Enterobacteriaceae.* Bifidobacteria*, Enterobacteriaceae, and total bacteria were enumerated in MC and in PMC, the latter supplemented with 5.0 E + 07 cfu/mL of* B. breve* B632 (Figures 1(a) and 1(b)). After 18 h of cultivation, FISH bacterial counts became steady in both MC and PMC cultures. Eubacteria increased up to 9.0–9.4 E + 09 cfu/mL, without statistically significant difference between PMC and MC (*P* > 0.05). At all the time points,* bifidobacteria* were more abundant in PMC than in MC (*P* < 0.05). Enterobacteriaceae were negatively affected by the presence of* B. breve* B632 and were always less numerous in PMC than in MC (*P* < 0.05).

**Figure 2:**
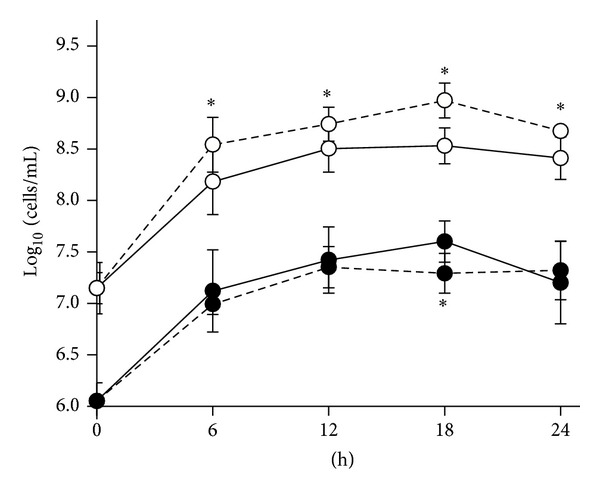
Time-course of* E. coli* and Enterobacteriaceae in cultures of infant gut microbiota.* E. coli* (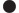) and Enterobacteriaceae (⚪) were quantified by qPCR in control cultures (MC, dashed line) and in cultures supplemented with* B. breve* B632 (PMC, solid line). Data are means ± SD, *n* = 4. Stars indicate statistically significant difference between MC and PMC cultures (*P* < 0.05).

The evolution of Enterobacteriaceae and* E. coli* was determined also with q-PCR during the whole process. Enterobacteriaceae were significantly lower in PMC than in MC (*P* < 0.05), consistently with FISH results. On the other hand, statistically significant difference was not observed in the levels of* E. coli* (*P* > 0.05), with the exception of 18 h, when* E. coli* was less numerous in MC than in PMC (Figure 2).

The presence of* B. breve* B632 in PMC cultures was traced using RAPD-PCR fingerprinting at all the time points. Colonies were isolated using the* Bifidobacterium* selective medium RB and those positive to* Bifidobacterium*-specific PCR were subjected to RAPD-PCR analysis. At the beginning of the fermentation,* B. breve* B632 represented the 85% of bifidobacterial isolates in PMC, then decreased to 73% after 6 h, and stabilized at 64% at the steady state (*n* = 4, SD < 34%). The relative amount of* B. breve* B632 tended to decrease, albeit differences at the diverse time points were not statistically significant. Considering that at the steady state Bifidobacteria accounted for approximately 38% of total eubacteria according to FISH enumeration,* B. breve* B632 can be estimated as approximately the 24% of total bacterial population in PMC. In these samples, 2 biotypes of* Bifidobacteria* represented the autochthonous component. The same two biotypes were identified also at the inoculum in MC cultures, together with two other minor ones, none of them exhibiting a RAPD-PCR profile similar to that of* B. breve* B632.

**Figure 3:**
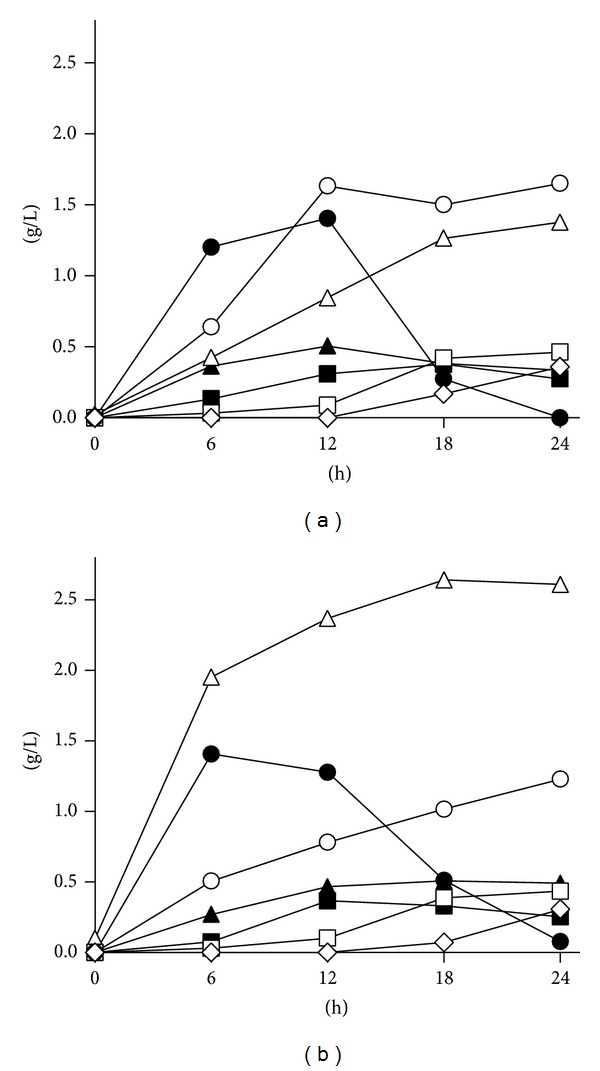
Time-course of fermentation products in cultures of infant gut microbiota. Ethanol (⚪), lactate (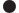), acetate (△), formate (▲), propionate (□), 2,3-butanediol (■), and butyrate (22C4) were determined in control cultures (MC, (a)) and in cultures supplemented with* B. breve* B632 (PMC, (b)). Data are means, *n* = 4, and SD always < 0.25 g/L.

Formate, acetate, lactate, propionate, butyrate, and ethanol originated by microbiota metabolism during the processes (Figures 3(a) and 3(b)). Like the bacterial counts, the concentrations of microbial products became stationary after approximately 18 h. Ethanol, formate, lactate, and acetate were the first to increase at the beginning of the fermentation. Propionate, 2,3-butanediol, and butyrate accumulated later, while lactate decreased as the steady state was approached.

During the growth phase, the major differences between MC and PMC processes were acetate and ethanol, accumulating at different levels during the first hours of the process: after 12 h, in MC and PMC, ethanol was 1.6 and 0.8 g/L, while acetate 0.8 and 2.4 g/L, respectively. At the steady state (18 h), MC had higher levels of butyrate and ethanol than PMC, while acetate and lactate were higher in PMC (*P* < 0.05). The other metabolites exhibited similar steady-state concentrations in PMC and MC processes (*P* > 0.05).

## 4. Discussion

Literature reports the use of* Lactobacillus* spp. strains to alleviate the symptoms of infant colic [22, 23]. On the other hand, no information is available on this specific use of* bifidobacteria*, although* in vitro* results showed that strains of* Bifidobacterium* can exert antimicrobial activity against gas forming coliforms [24]. Among a panel of* Bifidobacterium* strains that were selected as potential candidates for probiotic use against colic in infants,* B. breve* B632 appeared particularly promising because of its strong antimicrobial activity against coliforms, coupled to the lack of transmissible antibiotic resistance traits and cytotoxicity for the gut epithelium. Moreover, the strain is capable of adhering to gut epithelium cell lines and could stimulate gut health by increasing metabolic activity and immune response of epithelial cells [24].

In the present work, the antagonistic effect of* B. breve* B632 against coliforms was challenged within gut microbiota cultures of a colicky newborn, simulating* in vivo* conditions, in order to propose its use as anticolic probiotic.* B. breve* B632 survived well within the fecal culture, exhibiting a high viability during the process. At all the time points, Enterobacteriaceae were significantly less numerous in presence of the probiotic. These results indicate that* B. breve* B632 exerted antimicrobial activity against coliforms in fecal cultures as well, consistently with previous observation with spot agar tests and cocultures [24].

Unlike Enterobacteriaceae,* E. coli* counts were not affected by the presence of the probiotic. This observation can be ascribed to the different specificity of the primer sets utilized in qPCR quantification, since the primers for Enterobacteriaceae recognize a broader spectrum of species than the ones for* E. coli* (Table 1).

Based on the list of species that align with qPCR primers and FISH probes, it is likely that Gammaproteobacteria other than* E. coli* are involved in infant colic. For example, the qPCR primers for Enterobacteriaceae should recognize* Yersinia*, whereas the FISH probe for Enterobacteriaceae is expected to miss it.

Fecal samples have a microbial composition that does not exactly correspond to that of the colonic content, where major microbial-host interactions occur, and richness and diversity seem underrepresented [34]. However, systems as the one herein described are currently the best tools to investigate the external factors that could influence the intestinal microbial composition such as antibiotics or to test novel potential probiotics, before carrying out expensive* in vivo* trials. The data herein presented indicate that the potential probiotic strain* B. breve* B632 was able to survive in a complex microbial environment and restrained Enterobacteriaceae population.

## 5. Conclusions

The present study demonstrated the ability of a properly selected probiotic* Bifidobacterium* strain* B. breve* B632 to inhibit the growth of Enterobacteriaceae in an* in vitro* model system simulating the intestinal microbiota of a 2-month-old colicky infant. These results support the possibility to move to another level of study, that is, the administration of* B. breve* B632 to a cohort of colicky newborns, in order to observe the behavior of this strain* in vivo* and to validate its effect in colic treatment.

## Figures and Tables

**Table 1:** Genera of human intestinal bacteria potentially recognized by FISH probes and qPCR primers, according to SILVA.

Probe or primer set	Genus
Enterobact D	*Citrobacter *
	*Cronobacter *
	*Edwardsiella *
	*Enterobacter *
	*Escherichia *
	*Klebsiella *
	*Kluyvera *
	*Pantoea *
	*Raoultella *
	*Serratia *
	*Shigella *

Ent-F/Ent-R	*Edwardsiella *
	*Escherichia *
	*Klebsiella *
	*Pantoea *
	*Proteus *
	*Providencia *
	*Pseudomonas *
	*Shigella *
	*Yersinia *

Eco-F/Eco-R	*Cronobacter *
	*Escherichia *
	*Shigella *
